# Predicting patient post-detoxification engagement in 12-step groups with an extended version of the theory of planned behavior

**DOI:** 10.1186/s13722-015-0036-3

**Published:** 2015-06-20

**Authors:** John-Kåre Vederhus, Sarah E. Zemore, Jostein Rise, Thomas Clausen, Magnhild Høie

**Affiliations:** Addiction Unit, Sørlandet Hospital HF, PO Box 4164, 604 Kristiansand, Norway; Alcohol Research Group, Emeryville, CA USA; Norwegian Institute for Alcohol and Drug Research, Oslo, Norway; Norwegian Center for Addiction Research, University of Oslo, Oslo, Norway; Fulbright Scholar (2014–15), Alcohol Research Group, Emeryville, CA USA; University of Agder, Grimstad, Norway

**Keywords:** Theory of planned behavior, Alcoholics Anonymous, Detoxification treatment, Norway

## Abstract

**Introduction:**

Individuals with substance use disorders can receive important abstinence-specific support in 12-step groups (TSGs). However, our understanding of key factors that influence TSG participation remains limited. This study used an extended version of the theory of planned behavior (TPB) to enhance the understanding of TSG affiliation.

**Methods:**

Data were retrieved from a controlled trial of a 12-step facilitation intervention conducted on an inpatient detoxification ward in Norway (*N* = 140). Surveys at baseline included a TPB questionnaire. The behavioral target was to attend at least two TSG meetings per month in the 6-month follow-up period. Structural equation modeling was used to analyze the predictors of behavior at follow-up.

**Results:**

We found that attitudes, the moral norm, and perceived behavior control accounted for 81 % of the variance in the intention to participate regularly in TSGs after treatment. Subjective norms did not significantly influence the intention to participate. Moreover, the intention to participate significantly predicted behavior (β = 0.42, *p* < 0.001). In contrast to theory, there was a substantial, model-independent pathway from past to later behavior (β = 0.22, *p* = 0.047). The model explained 46 % (*p* < 0.001) of the variance in behavior. Attending ≥ 12 TSG meetings in the follow-up period was associated with a high percentage of abstinent days at follow-up (β = 0.38, *p* = 0.023).

**Conclusions:**

The present TPB questionnaire worked well for assessing patient intentions to attend a TSG. Treatment providers should encourage patient intentions to participate in TSGs post-detoxification.

**Electronic supplementary material:**

The online version of this article (doi:10.1186/s13722-015-0036-3) contains supplementary material, which is available to authorized users.

## Introduction

A central dimension of addiction is the individual’s ability to control his/her behavior in relation to use of the drug [1]. When patients with substance use disorders (SUDs) are in recovery, they must cope with triggers and urges over long periods of time, and they must develop self-regulation strategies to maintain rehabilitation. Thus, long-term support is essential [2]. Health services are encouraged to develop formal, continuing-care treatment efforts, but also to engage resources outside the health sector, including referrals to mutual-help groups [3, 4].

Mutual-help groups are one of the most widely available continuing-care options. The most common addiction-related groups are the 12-step groups (TSGs), which include Alcoholics Anonymous (AA) and Narcotics Anonymous (NA) [5]. TSGs can give attendees abstinence-specific support [6]. The teachings and practices of these groups can function as a cognitive antidote to the self-regulatory problems connected to the SUD; consequently, these groups may be able to impede the relapse process and contribute to maintaining remission [7, 8]. Thus, one way to understand the essence of addiction-related mutual help is to see it as a force that contrasts and countervails addiction [9]. Several studies have shown that treatment approaches aimed at connecting patients to abstinence-supportive peers have yielded better long-term outcomes than control conditions [10–12]. Consequently, affiliation with a TSG, defined as meeting attendance and involvement (e.g., having a sponsor, reading TSG literature), is currently considered a proximal treatment outcome [13, 14].

In considering TSG participation as a proximal outcome, it is important to understand the key influencing factors, particularly in countries where patients with SUDs are less likely to participate in TSGs [15]. Therefore, in the present study, we chose not to focus on patients’ intentions and perceived control in abstaining from substance use; instead, we focused on a possible antidote to addiction that might make achieving abstinence more likely: participation in a TSG. Most TSG studies have been conducted in the United States, where it is the norm to prescribe TSG participation in parallel with treatment [16]. Early studies about the TSGs were generally not guided by a theoretical framework, and they did not examine the most logical factors that influenced participation; i.e., TSG-specific beliefs and patient attitudes towards TSGs [17]. In the last decade, a few validated scales were developed based on theoretical frameworks. One of these scales, the Survey of Readiness for AA Participation (SYRAAP), was created in the framework of the Health Belief Model [18]. SYRAAP assesses the perceived severity of the alcohol problem and the perceived benefits and barriers to AA participation. Its composite score and the severity of the alcohol problem could predict later AA involvement [19].

Another important theoretical framework that has been used to explain TSG affiliation is the Theory of Planned Behavior (TPB) [20]. The TPB posits that behavioral performance results from reasoned deliberations mediated by the intention to perform a specific behavior [21]. Intention is predicted by the person’s *attitude*, defined as the positive or negative evaluation of the behavior in question; by the *subjective norm* (SN), defined as the perception of whether important others wish or expect the individual to behave in a certain way; and by *perceived behavioral control* (PBC), defined as the individual’s perception of how easy or difficult it is to execute the behavior. Because the principle of compatibility is important, all constructs must be defined in terms of exactly the same elements, and they must be specific about the target behavior (here, the target behavior was attendance to a TSG at least twice a month) and the time period (here, the time period was within the 6 months following detoxification) [21]. Intention can then be assumed to be the immediate antecedent of behavior.

According to the TPB, valid measures of the first-order TPB constructs (attitude, SN, and PBC) should sufficiently account for all meaningful variance in behavioral intention. Background factors, such as demographics, severity, and past behavior, should contribute as a function of these first-order constructs and not be added to explain the variance in intention [21]. Nonetheless, a meta-analysis of 12 datasets showed that, after taking into account the attitude, SN, and PBC, past behavior explained, on average, a further 7 % of the variance in intention [22]. In contrast to theory, a number of studies have also shown that past behavior can have a model-independent influence on later behavior. In the above-mentioned meta-analysis, past behavior explained a mean 13 % of the variance in behavior, after the TPB components had been accounted for [22].

Among the TPB constructs, SN was shown to be the weakest for predicting intention [23]. The definition of SN is said to be too narrow to capture all the aspects of normative influence. For example, SN does not take into account the individual’s personal beliefs of what is right and wrong [24]. One’s personal norm reflects an individual’s internalized moral rules or feelings of moral obligation, (hence, also called the *moral norm*), whereas SN only reflects the individual’s perceptions about what others would want him/her to do [24]. In a previous review, the moral norm was found to be a significant independent predictor in nine out of 10 studies, and it added an average 4 % to the prediction of intention [22]. Hence, moral norm was suggested to be an extended component of the TPB, especially relevant for TPB research in behavioral domains with moral or ethical dimensions [25, 26]. Thus, like the other first-order components, the moral norm should have an indirect impact on behavior by strengthening intention.

Only one previous study assessed TSG involvement in a TPB framework. It found that the TPB model could significantly predict both intention (R^2^ = .31) and behavior, measured as TSG affiliation; and the sum of TSG attendance and involvement (R^2^ = .41) [20]. To our knowledge, no prior study has used the TPB framework to examine TSG-related behavior outside the United States.

### Objectives

The aims of the present study were to: a) test the utility of the extended TPB model (including the moral norm) in predicting the intention to participate in a TSG after detoxification; and b) test whether intention predicted behavior. Based on previous findings in a British study, where patients with higher AA involvement at baseline were more likely to attend meetings after treatment [27], we also examined: c) whether the influence of past behavior on future behavior was mediated through TPB components. Finally, we examined: d) whether TSG attendance was associated with better substance use outcomes.

## Methods

### Sample and study setting

Participants were recruited from a detoxification department at the Addiction Unit, Sørlandet Hospital, in Kristiansand, Norway, between September 2008 and August 2010. The study was designed to test the efficacy of a motivational intervention to enhance post-treatment affiliation with TSGs, compared to brief advice that patients should attend a TSG. Patients were eligible for the study when discharge to their home was planned and they were not scheduled to receive additional inpatient or opioid maintenance treatment after detoxification. Exclusion criteria included severe psychiatric disorders or cognitive impairment. Of 156 eligible patients, 16 refused to participate, and the final sample included 140 patients (89 % of eligible respondents). A separate study examined the main trial outcomes and provided a detailed description of the sample, setting, and design [14]. The study was approved by the Regional Ethics Committee of the South-East Health Region, Norway.

### Measures

At baseline (Time 1), after providing informed consent, all participants completed the survey described below.

#### TPB questionnaire

A TPB questionnaire was developed based on Ajzen’s guidelines [28]. The target behavior, set by the researchers, was to attend at least two TSG meetings per month during the 6 months following detoxification. All model components were assessed with semantic, differential endpoints (e.g., “extremely unlikely” and “extremely likely”), and were rated on 7-point, unipolar, or bipolar scales. Intention to participate in TSGs was measured on a unipolar scale, with two items (e.g., “I intend to go regularly to AA/NA meetings [at least twice a month] over the next 6 months”). Direct measures of attitude toward TSG participation were assessed with six bipolar, adjective pairs (e.g., “worthless – valuable,” “unfavorable – favorable,” “pleasant – unpleasant”). The direct measure of SN was assessed on a bipolar scale, where participants rated whether people important to them would like them to attend TSGs (e.g., “People who are important to me think I should attend AA/NA meetings regularly [at least twice a month] over the next 6 months”). The direct measure of PBC was assessed with three unipolar items (e.g., the patient considered attending AA/NA meetings regularly over the next 6 months might be “easy” – “difficult”). The extended model included two questions rated on bipolar scales, which were related to the moral norm: “I would have a guilty conscience if I did not attend regular AA/NA meetings (at least twice a month) over the next 6 months,” and “It would be morally wrong of me if I did not attend regular AA/NA meetings (at least twice a month) over the next 6 months.” For the moral norm construct, we referred to nonaction rather than action, a commonly used method in previous research [26]. For the detailed questionnaire, see Additional file 1.

#### Alcoholic anonymous participation

Previous participation in TSGs was measured with the AA Affiliation Scale, modified to include both AA and NA [29]. For the present analysis, we used a dichotomized variable to indicate *past behavior;* patients indicated whether they had or had not ever attended TSG meetings prior to hospital admission.

#### Patient demographics and substance use

The Addiction Severity Index, European version (EuropASI), was used to collect data on patient demographics, life context, treatment history, and substance use [30, 31].

#### Outcome measures

At the 6-month follow-up after detoxification (Time 2), patients were re-interviewed. Of 140 patients, 113 completed the follow-up (81 %). A timeline follow-back technique was used to obtain the frequency of attendance (self-reported number of meetings per month of follow-up) [32]. A dichotomous variable (No = 0 and Yes = 1) was computed, based on the above data to indicate whether patients achieved the target behavior of regular attendance (attending TSGs at least twice a month) during the follow-up period (i.e., at least 12 meetings during follow-up). Attending 12 meetings was in itself not considered reaching the target behavior if the attendance was not regular. Ideally, the behavioral target could have been set to a more intensive level; a minimum of one weekly meeting has been recommended as an effective frequency in the literature [33]. However, clinical experience informed us that participation frequency and meeting availability was usually lower in Norway than in countries where TSGs are more common [34]. Hence, we set a lower target behavior threshold. Substance use outcomes were assessed with the EuropASI. Drug and alcohol use in the 30 days preceding the follow-up interview were evaluated to determine the percentage of days abstinent (PDA).

### Statistical analyses

Descriptive statistics are shown for baseline variables. A structural equation modeling analysis was performed, where the items described in the TPB questionnaire were used as indicators for the latent variables: attitude, SN, PBC, moral norm, and intention. We examined whether these factors predicted behavior at the 6-month follow-up, and whether the effect of prior behavior on later behavior was fully mediated by TPB components, as hypothesized by Fishbein & Ajzen [21]. We allowed for correlation between latent, first-order TPB constructs, according to the theory [21]. We controlled for the condition assignment (motivational intervention or brief advice) to account for the experimental design of the main study. Finally, we examined whether behavior predicted the outcomes of improved substance use. Due to the dichotomous outcome variable (behavior), we estimated these predictions with a weighted least-squares analysis, with correction of means and variances. The root mean square error of approximation (RMSEA; cutoff ≤ 0.05) and the comparative fit index (CFI; cutoff ≥ 0.95) were used as global fit measures [35]. Results are reported as fully standardized estimates, β and R-square (R^2^). The analysis was conducted using Mplus software, version 7.2. To handle missing values, we used the default procedure in Mplus, with full information maximum likelihood. The significance level was set at *p* < 0.05.

## Results

The sample was a mixed population with either an alcohol and/or a drug use disorder. Patients had > 11 years of problematic use of their major substance of abuse; 29 % had used an injected drug in the 6 months prior to admission (Table 1). Seventy-seven percent had received prior specialized SUD treatment, and nearly half (48 %) had previously participated in at least one TSG meeting.

**Table 1:** Characteristics of study respondents (*N* = 140)

Characteristic	N (%) or Mean (SD)
Age, years	41 (14)
Female	45 (32)
Proportion native Norwegians or European origin	134 (96)
Education, years	11.2 (2.3)
Relationship, proportion of singles	66 (47)
Main diagnosis (ICD-10)	
(1) Alcohol dependence (*N* = 48) or harmful alcohol use (*N* = 6)	54 (39)
(2) Both alcohol and drug dependence	26 (19)
(3) Drug dependence	60 (43)
Years of problematic use^a^ of the major drugs of abuse	11.4 (9.0)
Injection use in the last 6 months	40 (29)
Earlier SUD treatment (prior to current detoxification)	108 (77)
Ever participated in TSGs before	67 (48)

^a^ Problematic use, as defined in EuropASI, was the consumption of 5 or more standard drinks at least 3 times weekly, or binge drinking on 2 consecutive days to a level that affected daily functioning. For drug use, only frequency was needed; 3 times weekly or 2 consecutive days

The scores on TPB components were grouped into equal thirds to examine distribution for the low (LOW), middle (MOD), and high ends (HIGH) of the scale (Table 2). Intention, PBC, and the moral norm were distributed in roughly equal thirds, but attitudes and SN were skewed to the more positive end of the scale.

**Table 2:** Descriptive presentation of TPB components at baseline, expressed as the summed scores distributed into thirds^a^ (*N* = 140)

	Mean (SD)	LOW (%)	MOD (%)	HIGH (%)
Behavioral intention ^b^	4.2 (2.4)	31	29	39
Attitudes ^c^	1.2 (1.7)	10	29	61
SN ^c^	1.2 (1.7)	9	39	52
PBC ^b^	4.1 (1.9)	31	33	36
Moral norm ^c^	0.4 (2.1)	27	31	41

^a^ Seven-point ordinal scales consisted of 6 steps; each two steps comprised one-third of the scale. For example, for a unipolar scale, the lower third (LOW) = 1–3, mid-third (MOD) = 3.01–5, and the highest, most positive third (HIGH) = 5.01–7. The full scale was used in the analyses

^b^ Unipolar scale (1 to 7; 4 is the scale midpoint)

^c^ Bipolar scale (−3 to 3; 0 is the scale midpoint)

### Latent path analysis

**Fig. 1:**
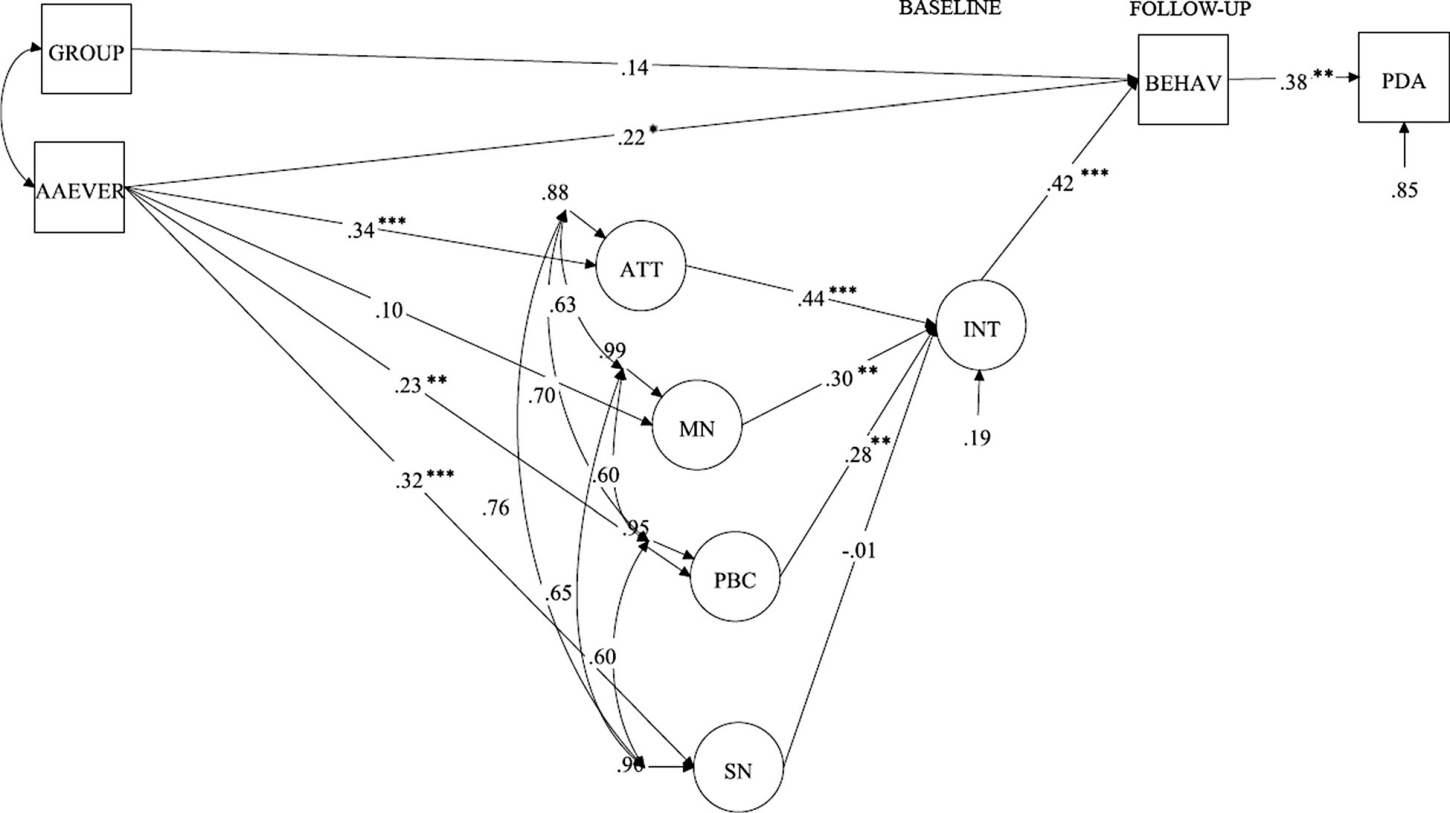
Latent path analysis predicting regular attendance of patients with SUD in a TSG after detox treatment. The structural model is presented with standardized factor loadings. Notes: Significant paths are marked with: * = <0.05, ** = <0.01, *** = <0.001. Abbreviations: BEHAV = behavior at 6 month follow-up, AAEVER = any prior attendance in a TSG; GROUP = condition assignment (factor loading for the motivational intervention is shown), ATT = Attitudes, SN = subjective norms, MN = moral norm, PBC = perceived behavioral control, INT = Intention, PDA = percentage of days abstinent

**Fig. 2:**
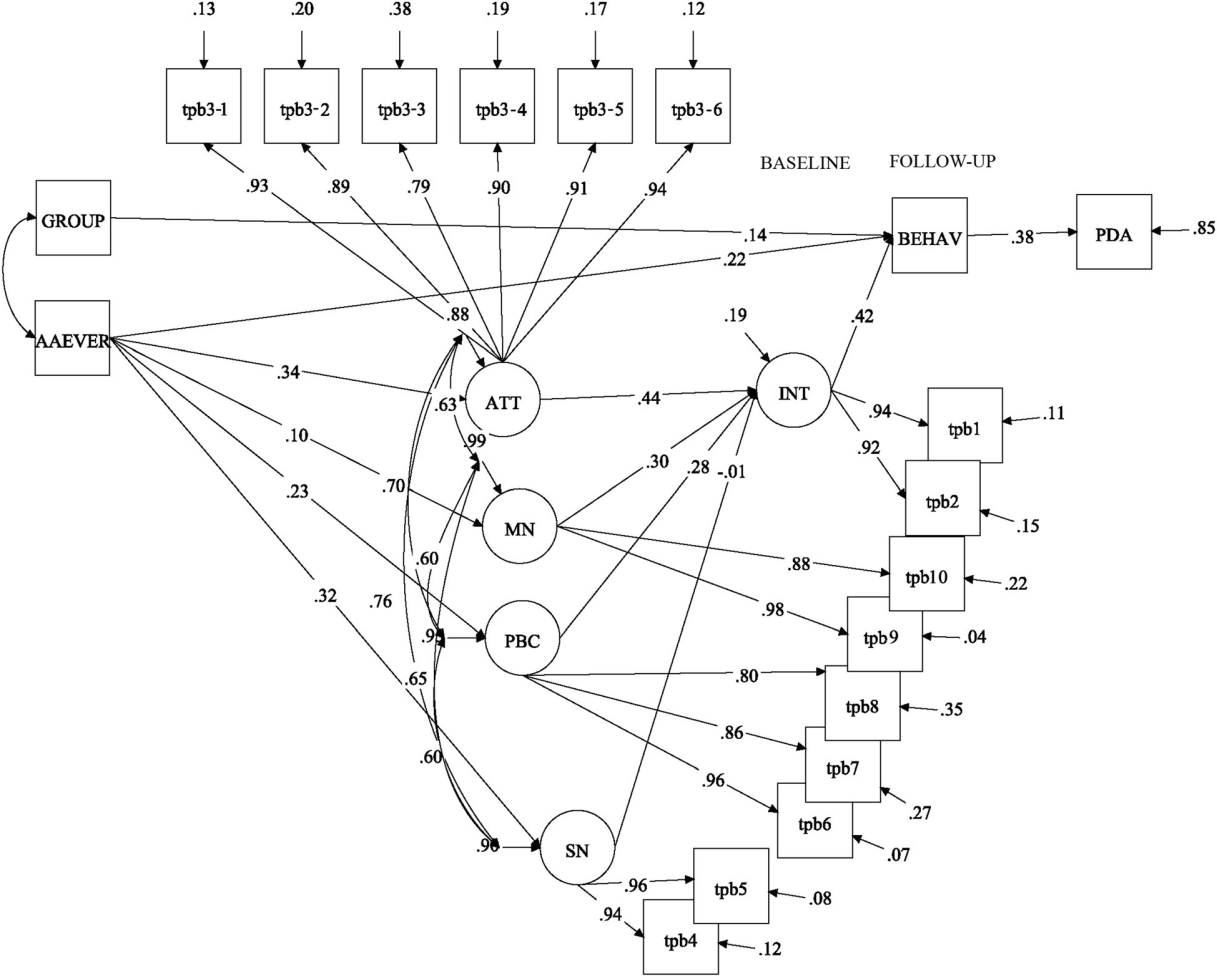
Latent path analysis predicting regular attendance of patients with SUD in a TSG after detox treatment. The full path diagram, including the measurement model, is shown with standardized factor loadings. Notes: All factor indicator paths were significant at the *p* <0.001 level. For significance level of structural paths, see Fig. 1. Abbreviations: BEHAV = behavior at 6-month follow-up, AAEVER = any prior attendance in a TSG; GROUP = condition assignment (factor loading for the motivational intervention is shown), ATT = Attitudes, SN = subjective norms, MN = moral norm, PBC = perceived behavioral control, INT = Intention, PDA = percentage of days abstinent, TPB1–10 = TPB questionnaire item number (see Additional file 1 “TPB measure” for description)

For simplicity, only the structural TPB model is shown in Fig. 1. The full path diagram, including the measurement model, is shown in Fig. 2. The global fit of the model was excellent, with an RMSEA = 0.02 and CFI = 0.98; the model was positively identified with a *χ*^2^ = 147.0, df = 137, and *p* = 0.264. Attitude (β = 0.44, *p* < 0.001), moral norm (β = 0.30, *p* = 0.001), and PBC (β = 0.28, *p* = 0.004), but not SN (β = −0.01, *p* = 0.94), were significant predictors of intention. Past behavior significantly influenced attitude, SN, and PBC, but not moral norm. A preliminary analysis (second-order confirmatory factor analysis) showed that past behavior had no direct effect on intention (β = 0.03, *p* = 0.603); thus, the influence of past behavior on intention was fully mediated by the first-order TPB components. To facilitate identification of the full model, this nonsignificant path was omitted in the final analysis (Fig. 1).

At the follow-up, 32 patients (28 %) achieved the behavioral goal of attending at least two meetings each month during the follow-up period. Intention significantly predicted behavior (β = 0.42, *p* < 0.001; Fig. 1). The total indirect effect of past behavior upon later behavior (via TPB components) was β = 0.11, *p* = 0.012. However, past behavior also had a substantial, independent effect on later behavior (β = 0.22, *p* = 0.047). Thus, the influence of past behavior upon later behavior was only partially mediated by TPB components. The condition assignment (motivational intervention or brief advice) did not fully explain the variance in the defined behavior target in this analysis (β = 0.14, *p* = 0.21; Fig. 1). The full model explained 81 % (*p* < 0.001) of the variance in intentions and 46 % (*p* < 0.001) of the variance in behavior (R^2^).

In turn, attending at least 12 TSG meetings in the follow-up period was associated with a higher PDA at follow-up (β = 0.38, *p* = 0.023) and explained 15 % of the variance in PDA. Those attending at least 12 TSG meetings had a mean of 89 % (SD = 25, 95 % CI = 80 %–98 %) abstinent days, compared to 62 % (SD = 43, 95 % CI = 52 %–71 %) for those with lower attendance rates.

## Discussion

Attitudes, moral norm, and PBC, but not SN, were significant determinants of the intention to attend at least two monthly TSG meetings in the follow-up period. In turn, intention significantly predicted behavior. Past behavior was fully mediated by the first-order TPB components in explaining variance in intention. However, there was a significant path from past behavior to later behavior that was not mediated by TPB components. Attending ≥ 12 TSG meetings was associated with higher PDA at follow-up.

The present study showed that the TPB questionnaire substantially facilitated the identification of factors that could explain the intention to participate in TSGs after detoxification. The first-order TPB components, including moral norm, explained more than 80 % of the variance in intention. Even in these relatively naïve 12-step settings, where only half of the sample had previous TSG exposure, a majority of the patients scored in the “high,” more positive, end of the attitude indicator scales [36]. This finding suggested that patients understood the potential benefits of TSG involvement. The attitude component also had the strongest impact on intention to attend groups regularly after detoxification. Furthermore, our findings suggest that attitudes were positively influenced by prior exposure to TSGs (past behavior).

The moral norm was the second strongest predictor of intention. In a previous review, it was suggested that the moral norm would primarily be relevant for TPB research in behavioral domains with moral or ethical dimensions [25]. Our findings indicated that the addiction field may be such a domain. Although the perceived external pressure from important others did not influence intention, there appeared to be an inherent obligation to act in a way that might mitigate the SUD. This perceived obligation did not seem to be connected to TSGs in particular, because past TSG participation did not significantly influence the moral norm. Rather, it may have been related to participation in generally available recovery activities.

SN was the weakest factor; it did not significantly predict intention. In contrast, a previous TPB study based in the U.S. found that SN was the strongest predictor of intention [20]. To some extent, this discrepancy may be due to the difference in familiarity with TSGs between the United States and Norway. TSGs are less extensively known in Norwegian society as a whole [15, 34]. However, in our study, there was no “floor effect” in the distribution of SN scores; the scores were skewed to the positive side of the scale. Thus, although important others were perceived to have a generally positive attitude towards TSGs, their opinions about attendance may not have been as strong as those of the important others mentioned in the U.S. sample [20]. Hence, in the present settings, addressing the SN may not be the most effective way to influence patient intentions.

The results, which are consistent with the TPB, imply that various behavior-change strategies may be effective for clinicians aiming to enhance participation in TSGs [21]. For example, a clinician could seek to enhance a patient’s PBC over TSG involvement by pairing him or her with someone who is affiliated with the group; this volunteer could then provide practical help in attending meetings (e.g., help with selecting a specific meeting, information on the meeting protocol, and a ride). This strategy is supported by prior studies that have found significantly higher TSG involvement among patients who were directly linked to AA volunteers who then offered to accompany them to a meeting [12, 37]. A second strategy is to motivate individuals with less positive attitudes by shifting their attitudes in a more positive direction. To do this, a clinician could highlight the potential benefits of participation, such as the potential to make new friends who are living clean and sober lifestyles. The clinician might also explore the patient’s concerns about TSGs and possible negative outcomes of participation. For example, some patients may be concerned that they will be stigmatized if they do not share the religious or spiritual beliefs of TSG members, and/or (incorrectly) believe that TSGs proscribe the use of all medically prescribed drugs [38]. A clinician who is familiar with TSGs could help to dispel these fears by, for example, pointing to the vast diversity in concepts of a higher power among TSG members, as well as by referring the client to TSG literature (e.g., pamphlets explaining AA/NA concepts and practices) [39]. Third, individuals who have already formed positive attitudes, have high perceived control over involvement, and who intend to become involved could be encouraged to act on their intentions. One way to encourage patients with this profile is to help them formulate the implementation plan for their intentions; this strategy has resulted in a significantly improved translation of intentions into action [40]. To formulate the implementation plan, one must outline with the patient the intention in a more detailed, practical manner, and think through the when, where, and how of future actions [41]. A patient could thus be encouraged to plan a specific schedule of meeting attendance along with, perhaps, activities for each meeting (e.g., introducing oneself to a member, approaching a potential sponsor).

The intention-behavior relationship was significant according to theory, and the amount of explained variance in behavior was surprisingly strong in the final model. However, this strong explanatory feature was due, not only to TPB constructs, but also to the inclusion of a background factor, past behavior. One might be skeptical about a patient’s stated intention in the sheltered environment of a detoxification ward, but our findings showed that intention accounted for an appreciable variance in behavior, even in these settings. Behavior was also significantly associated with improved substance use outcomes. Thus, our findings were promising. They indicated that by targeting TPB-related components in these settings (e.g., attitude factors, behavior control, and perceived moral obligations; see practical and evidence-based examples above), we may be able to increase intention, and subsequently, increase the rates of regular TSG participation.

In previous European TSG research, higher AA involvement at baseline was found to predict higher levels of later participation. A British study found that, for each additional point on an AA involvement scale at baseline, a participant was twice as likely (OR, 2.3) to attend meetings after treatment [27]. Several of the findings in the present study corroborated this prior result. For example, we found that past TSG exposure positively influenced attitudes and PBC and that past behavior accounted for a substantial part of the variance in later behavior, even when intention was accounted for simultaneously. These findings indicated that, in European settings, it may be easier to move prior participants towards more TSG involvement than to engage newcomers; thus, it is likely that different strategies are needed for the two groups (see suggested strategies above). The TPB questionnaire presented here can be used clinically to guide clinicians in choosing between these strategies.

A previous study showed that clinicians in the region had moderately positive attitudes towards TSGs, but they were not associated with TSG engagement strategies on a regular basis [34]. Thus, a prerequisite for enhancing patient involvement in TSGs may be to work on clinicians’ attitudes. Since that study, initiatives have been made to improve the attitudes of clinicians in the region. We have surmised that there is growing interest in facilitating patient attendance in TSGs.

In the main report of this study, we showed that the group assigned to the motivational intervention had a significantly favorable effect on the primary outcome, which was a combined score of TSG attendance and involvement. In contrast, its effect on attendance alone, a secondary outcome, was borderline significant [14]. In the present investigation, the behavioral target was a certain level of regular attendance (≥12 meetings during follow-up), and we found that group assignment (motivational intervention or brief advice) was not a significant factor. This lack of effect suggested that the group effect was partly mediated by variables in the model, or alternatively, that the motivational intervention was not strong enough to detect a significant effect on the number of respondents who achieved this level of regular attendance.

### Methodological considerations

This study was one of the few to examine TSG-related behavior among patients in a non-U.S. setting, and it was one of the few to investigate this specific type of behavior (mutual-help group participation) in a TPB framework. The TPB model is a causal model; hence, it has explanatory power. Despite the fact that past behavior had an independent effect (beyond intention) in the present study, one cannot necessarily draw the conclusion that a predictor like past behavior has a causal effect on later behavior [21, 42].

The present findings must be interpreted in the context of certain study limitations, such as the age of the data, which are 5 years old, and reliance on self-report. TSGs do not have records of attendees. Thus, the only possible way to study this phenomenon is through respondent self-report [43]. Moreover, PDA was a secondary outcome that is not a part of the original TPB model. Although experimental trials of 12-step facilitation do suggest a causal association between involvement in TSGs and higher odds of abstinence, we cannot establish with the present study that the path from behavior (i.e., TSG attendance) to PDA is necessarily causal [44]. In addition, the relatively small sample size (*N* = 140 subjects) and estimation of multiple models may lead to the instability of parameter estimates or problems with multiplicity.

### Implications

The improved substance use outcomes among regular TSG attendees demonstrated that treatment providers should support and encourage a patient’s intention to participate in TSGs after detoxification. The TPB questionnaire presented here can be used clinically to guide clinicians in this type of treatment work.

## Conclusions

The present TPB model worked well in accounting for respondents’ self-reported TSG attendance. Moreover, our model identified factors that significantly predicted later behavior. Regular TSG attendees had a higher PDA than nonregular attendees at follow-up. Our findings have shed light on the process of becoming involved in TSGs and offered suggestions on how treatment providers can promote patient TSG involvement.

## Additional file

Additional file 1:
**Theory of Planned Behavior measure.**

## Abbreviations

AA: Alcoholics Anonymous
CFI: Comparative fit index
NA: Narcotics Anonymous
PBC: Perceived behavior control
PDA: Percentage of days abstinent
RMSEA: Root mean square error of approximation
SN: Subjective norm
SUD: Substance use disorders
TPB: Theory of planned behavior
TSG: 12-Step group
