# Supplementary material for: Predicting patient post-detoxification engagement in 12-step groups with an extended version of the theory of planned behavior
Source: Addict Sci Clin Pract. 2015 Jun 20;10:15. doi: 10.1186/s13722-015-0036-3 (PMC4636789; doi:10.1186/s13722-015-0036-3)
Supplement: Additional file 1: — Theory of Planned Behavior measure. [file 13722_2015_36_MOESM1_ESM.doc]

One measure which may help if you have a dependence problem is to participate in the self-help groups Alcoholics Anonymous (AA) or Narcotics Anonymous (NA)

Please indicate on each line the thoughts you have about FUTURE participation in AA/NA after the detox treatment. Some of these questions may seem similar, but please answer ALL of them. We want your personal opinion; consequently, there is no “right” or “wrong” answer.

| Please give your opinion on the following assertions about your thoughts about the future: |
| --- |

| 1. I intend to go regularly attend AA/NA meetings (at least twice a month) over the next six months. |
| --- |

| Definitely false | 1 | 2 | 3 | 4 | 5 | 6 | 7 | Definitely true |
| --- | --- | --- | --- | --- | --- | --- | --- | --- |

| 2. I will attend regular AA/NA meetings (at least twice a month) over the next six months: |
| --- |

| Highly unlikely | 1 | 2 | 3 | 4 | 5 | 6 | 7 | Highly likely |
| --- | --- | --- | --- | --- | --- | --- | --- | --- |

| To what extent do you agree or disagree with the following statements: |
| --- |

| 3. For me, attending regular AA/NA meetings (at least twice a month) over the next six months will be: |
| --- |

| Worthless | -3 | -2 | -1 | 0 | 1 | 2 | 3 | Valuable |
| --- | --- | --- | --- | --- | --- | --- | --- | --- |
| Unreasonable | -3 | -2 | -1 | 0 | 1 | 2 | 3 | Reasonable |
| Unpleasant | -3 | -2 | -1 | 0 | 1 | 2 | 3 | Pleasant |
| Negative | -3 | -2 | -1 | 0 | 1 | 2 | 3 | Positive |
| Unfavourable | -3 | -2 | -1 | 0 | 1 | 2 | 3 | Favourable |
| Bad | -3 | -2 | -1 | 0 | 1 | 2 | 3 | Good |

| 4. People who are important to me think I should attend regular AA/NA meetings (at least twice a month) over the next six months: |
| --- |

| Strongly disagree | -3 | -2 | -1 | 0 | 1 | 2 | 3 | Strongly agree |
| --- | --- | --- | --- | --- | --- | --- | --- | --- |

| 5. People who are important to me feel that I should attend regular AA/NA meetings (at least twice a month) over the next six months: |
| --- |

| Strongly disagree | -3 | -2 | -1 | 0 | 1 | 2 | 3 | Strongly agree |
| --- | --- | --- | --- | --- | --- | --- | --- | --- |

| Give your view on the following: |
| --- |

| 6. For me, attending regular AA/NA meetings (at least twice a month) over the next six months will be: |
| --- |

| Difficult | 1 | 2 | 3 | 4 | 5 | 6 | 7 | Easy |
| --- | --- | --- | --- | --- | --- | --- | --- | --- |

| 7. Given that you wanted to attend regular AA/NA meetings (at least twice a month) over the next six months, how confident are you in your being able to do that: |
| --- |

| Very little confident | 1 | 2 | 3 | 4 | 5 | 6 | 7 | Very confident |
| --- | --- | --- | --- | --- | --- | --- | --- | --- |

| 8. How much control do you feel you have in terms of attending regular AA/NA meetings (at least twice a month) over the next six months: |
| --- |

| No control | 1 | 2 | 3 | 4 | 5 | 6 | 7 | Complete control |
| --- | --- | --- | --- | --- | --- | --- | --- | --- |

| To what extent do you agree or disagree with the following statements: |
| --- |

| 9. I would have a guilty conscience if I did NOT attend regular AA/NA meetings (at least twice a month) over the next six months: |
| --- |

| Strongly disagree | -3 | -2 | -1 | 0 | 1 | 2 | 3 | Strongly agree |
| --- | --- | --- | --- | --- | --- | --- | --- | --- |

| 10. It would be morally wrong of me if I did NOT attend regular AA/NA meetings (at least twice a month) over the next six months: |
| --- |

| Strongly disagree | -3 | -2 | -1 | 0 | 1 | 2 | 3 | Strongly agree |
| --- | --- | --- | --- | --- | --- | --- | --- | --- |
